# Mechanical versus manual cardiopulmonary resuscitation (CPR): an umbrella review of contemporary systematic reviews and more

**DOI:** 10.1186/s13054-024-05037-4

**Published:** 2024-07-30

**Authors:** Ayman El-Menyar, Mashhood Naduvilekandy, Sandro Rizoli, Salvatore Di Somma, Basar Cander, Sagar Galwankar, Fatimah Lateef, Mohamed Alwi Abdul Rahman, Prabath Nanayakkara, Hassan Al-Thani

**Affiliations:** 1 Clinical Research, Trauma and Vascular Surgery, Hamad Medical Corporation, Doha, Qatar; 2 Department of Medicine, Weill Cornell Medical School, Doha, Qatar; 3 Trauma Surgery, Hamad Medical Corporation, Doha, Qatar; 4 Postgraduate School of Emergency Medicine, Faculty of Medicine and Psychology, University La Sapienza Rome, Rome, Italy; 5 Emergency Medicine, Bezmialem Vakıf Üniversitesi, Istanbul, Türkiye; 6 Florida State University, College of Medicine, Emergency Medicine Residency Program, Sarasota Memorial Hospital, Sarasota, Florida, USA; 7 Department of Emergency Medicine, Singapore General Hospital, Singapore, 169608 Singapore; 8 Emergency Medicine, Trauma and Disaster Medicine, MAHSA University, Petaling jaya, Selangor, Malaysia; 9 General Internal Medicine, Amsterdam Public Health Research Institute, Amsterdam University Medical Centers, Amsterdam, The Netherlands; 10 Trauma and Vascular Surgery, Hamad Medical Corporation, Doha, Qatar

**Keywords:** CPR, ROSC, Mechanical CPR, Manual CPR, Survival, Cardiac arrest, OHCA, IHCA

## Abstract

**Background:**

High-quality cardiopulmonary resuscitation (CPR) can restore spontaneous circulation (ROSC) and neurological function and save lives. We conducted an umbrella review, including previously published systematic reviews (SRs), that compared mechanical and manual CPR; after that, we performed a new SR of the original studies that were not included after the last published SR to provide a panoramic view of the existing evidence on the effectiveness of CPR methods.

**Methods:**

PubMed, EMBASE, and Medline were searched, including English in-hospital (IHCA) and out-of-hospital cardiac arrest (OHCA) SRs, and comparing mechanical versus manual CPR. A Measurement Tool to Assess Systematic Reviews (AMSTAR-2) and GRADE were used to assess the quality of included SRs/studies. We included both IHCA and OHCA, which compared mechanical and manual CPR. We analyzed at least one of the outcomes of interest, including ROSC, survival to hospital admission, survival to hospital discharge, 30-day survival, and survival to hospital discharge with good neurological function. Furthermore, subgroup analyses were performed for age, gender, initial rhythm, arrest location, and type of CPR devices.

**Results:**

We identified 249 potentially relevant records, of which 238 were excluded. Eleven SRs were analyzed in the Umbrella review (January 2014–March 2022). Furthermore, for a new, additional SR, we identified eight eligible studies (not included in any prior SR) for an in-depth analysis between April 1, 2021, and February 15, 2024. The higher chances of using mechanical CPR for male patients were significantly observed in three studies. Two studies showed that younger patients received more mechanical treatment than older patients. However, studies did not comment on the outcomes based on the patient's gender or age. Most SRs and studies were of low to moderate quality. The pooled findings did not show the superiority of mechanical compared to manual CPR except in a few selected subgroups.

**Conclusions:**

Given the significant heterogeneity and methodological limitations of the included studies and SRs, our findings do not provide definitive evidence to support the superiority of mechanical CPR over manual CPR**.** However, mechanical CPR can serve better where high-quality manual CPR cannot be performed in selected situations.

**Supplementary Information:**

The online version contains supplementary material available at 10.1186/s13054-024-05037-4.

## Introduction

The incidence of cardiac arrest (CA) differs significantly worldwide, with some centers reporting up to ten times higher numbers than others. The average global incidence of out-of-hospital CA (OHCA) is estimated to be 55 per 100,000 persons per year [1]. Although there is a noticeable improvement in the utility of cardiopulmonary resuscitation (CPR), poor outcomes remain challenging. The in-hospital CA arrest (IHCA) in the USA occurs as 6–7 CAs per 1000 hospital admissions in contrast to 1.5–2.8 per 1000 in Europe, with a survival rate of 15–34% at hospital discharge or 30 days [2]. However, the overall bystander CPR in Europe is documented in only 58% of the OHCA settings (13–83%). One study from Sweden reported that resuscitation was attempted in only 12% of the IHCA [3]. The restoration of spontaneous circulation (ROSC) after CPR varies from 36 to 54% [2]. In some studies, the men-to-women incidence of IHCA is estimated as 1.4–1.6 to 1 [4]. The survival rate after OHCA is nearly 3–6% in Asia, 8% in Europe (0–18%), 11% in the USA, and 12% in Australia [2]. The annual incidence of OHCA in Europe is 67–170 per 100,000 persons [2]. Generally, the short-term survival rate after OHCA is 10–15% (one-tenth) in contrast to 20–25% (one-quarter) after IHCA [5–7]. The survival rate heterogeneity could be related to several factors, namely the gender, etiology of arrest, initial cardiac rhythm during CPR, time elapsed before CPR, location of cardiac arrest, and patient’s ethnicity [2]. Therefore, high-quality CPR is required to attain the ROSC, preserve brain perfusion and function, and save lives [7].

The basic principle of CPR requires manual chest compressions by bystanders and medical personnel. In recent decades, mechanical chest compression devices have been introduced, and their use is growing. Different types of mechanical CPR devices are available commercially. The two most common mechanical CPR devices are Load-Distributing Band (LDB), which offers circumferential thoracic compressions, and Piston-Driven (PD), which provides sternal compressions. The AutoPulse device (manufactured by ZOLL Medical Corporation, Chelmsford, Massachusetts, USA) and the LUCAS (Lund University Cardiopulmonary Assist Device) are typical examples of LDB and PD-CPR devices, respectively [8, 9]. Thumper and Lifestat, manufactured by Michigan Instruments, USA, are also PD-CPR devices [10]. The pneumatic vest is another CPR device that rapidly introduces air for chest compression [11].

Many studies have been conducted in the last two decades comparing manual and mechanical CPR, particularly after the introduction of the LUCAS and AutoPulse devices [12–16], including numerous systematic reviews (SRs) with conflicting conclusions [17–27]. There is no published umbrella review (UR) comparing the mechanical vs manual CPR and summarizing the recently published SRs. We aimed to assess the certainty of evidence published on the subject by identifying and summarizing the existing SRs in an umbrella review, focusing on the essential findings and methodologies, and identifying their consensus and discrepancies. We also added a new SR, including the original studies conducted after the last published SR, to bridge the gap and elucidate the latest developments in the field. To comprehensively assess the effectiveness of mechanical CPR over manual CPR, we include different subanalyses for study types, settings, devices, and outcome measures.

## Methodology

### Literature search for the umbrella review (UR)

Two authors (MNK & AE) independently searched PubMed, EMBASE, and Medline databases for published SRs between January 15, 2014, and February 15, 2024 (the study's timeline is given in the Suppl file). The last decade was selected to align with current practices in the field. Although we did not set language restrictions, all the used SRs were published in English. The comprehensive search strategy can be found in Appendix file.

### Inclusion and exclusion criteria

**Fig. 1:**
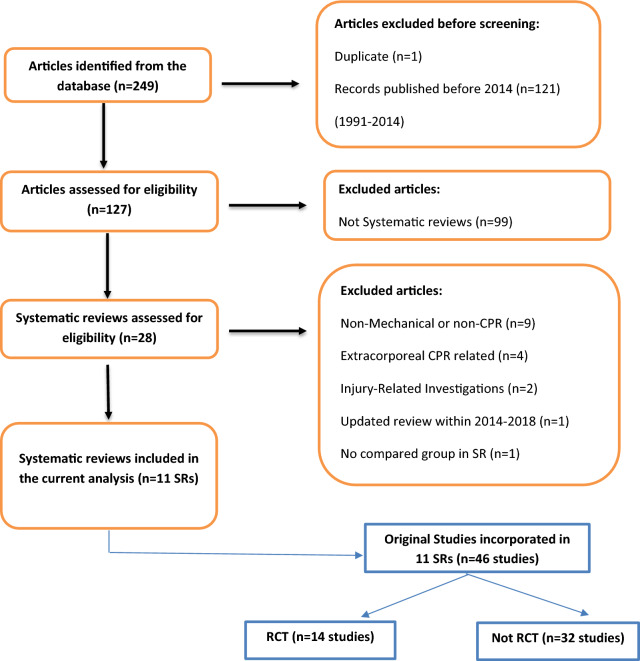
PRISMA flow chart for a systematic review of systematic reviews (up to February 2024)

We included the IHCA and OHCA studies and SRs that used randomized controlled trials (RCTs) and non-RCTs. This approach allowed for a wide range of evidence, methodology, and perspectives in the literature. Trentino et al*.* [28] concluded that it is essential not only to consider the study method but also the critical elements of the study design. We considered studies on different mechanical CPR devices (LBD and PD) and different outcome measures. Even though the inclusion of these distinct populations, study designs, devices, and outcome measures create heterogeneity, it is necessary for a comprehensive analysis of the topic and its overall effectiveness. Exclusion criteria are given in Fig. 1.

We aimed to include every SR published from January 15, 2014, to February 15, 2024, comparing mechanical to manual CPR. We excluded records that were not SRs (e.g., narrative reviews, case reports), did not compare mechanical with manual CPR, or included pediatric patients. Thereafter, we performed an up-to-date SR of the studies published after the last published SR.

#### PICO (population, intervention, comparison, outcome)

The population of interest was cardiac arrest patients, both IHCA and OHCA. The intervention groups consisted of patients who received mechanical or manual CPR. The outcomes of interest included one or more of the following: ROSC, survival to hospital admission, survival to hospital discharge, 30-day survival, and survival to hospital discharge with good neurological function.

The objectives of our work included (1) identifying and summarizing existing SRs (UR) to focus on the essential findings and methodologies, (2) identifying the consensus, discrepancies, and the effectiveness of mechanical versus manual CPR, (3) performing a new SR of the studies that were not included in the latest published SR, (4) performing subgroup analyses including age, gender, initial rhythm, type of device, and location of CA (IHCA and OHCA). These sub-analyses will include only studies that explicitly mention the number of patients, events, outcomes, and type of CPR device.

### Selection of studies and data extraction

Only SRs were included in the UR. Initially, MNK and AE selected studies independently by examining the title, abstracts, and full text. The same authors did the data extraction and quality assessment independently using the AMSTAR-2 tool, and any discrepancies were discussed and clarified. Information extracted from the full texts included study year, duration of search, intervention (LUCAS, AutoPulse, or other mechanical CPR), number of articles by study settings, sample size, risk of bias tools, study design, heterogeneity, outcomes measured, and conclusion.

### Quality assessment

To assess the methodological quality of included SRs, we used the Measurement Tool to Assess Systematic Reviews (AMSTAR-2). The AMSTAR-2 comprises 16 questions with answers: ‘Yes,’ ‘No,’ or ‘Partial yes.’ A complete guide on how to use and interpret the tool is available for users [29]. The overall confidence in the result of the review can be categorized into ‘High,’ ‘Moderate,’ ‘Low’ or ‘Critically low’ based on the seven critical domains out of the 16 (items 2, 4, 7, 9, 11, 13, and 15).

### New systematic review

We also conducted an additional SR analyzing all original studies that were published between April 2021 and February 15, 2024. Data extracted included RCTs, retrospective or prospective studies, study settings, country, study duration and quality, interventions, sample size, heterogeneity, and outcomes. The quality of the studies was assessed using the Newcastle–Ottawa Scale (NOS) [30]. Additionally, Grading of Recommendations Assessment, Development, and Evaluation (GRADE) was used to determine the overall certainty of evidence for each outcome [31].

*Protocol Registration* Our UR and SR protocol was registered a priori in the International Prospective Register of Systematic Reviews (*PROSPERO ID: CRD42024537182*).

## Results

### Results of the umbrella review (UR)

We identified 249 potentially relevant records; however, 238 were excluded (Fig. 1). Eleven eligible SRs (published between January 2014 and March 2022) were selected for the final data extraction. Excluded references are available in Appendix file.

### Characteristics of systematic reviews

The UR included 11 SRs involving original studies conducted between 1978 and March 2021 (Tables 1, 2). Wang et al. published two SRs, one in 2014 and another in 2018 [26]; we included the latter SR only. Ten SRs included original studies with different types of mechanical CPR devices. However, Liu et al. [18] included only studies using the LUCAS device, minimizing heterogeneity. One SR included only IHCA studies [21]; three used a combination of IHCA and OHCA [19, 20, 26], while seven SRs considered OHCA studies only. Most SRs reported high heterogeneity between the studies. Wang et al. [26] did not conduct a meta-analysis pointing to the high heterogeneity (> 50%) between the studies.

**Table 1:** Characteristics of the systematic reviews (Umbrella review)

References/duration	Intervention	No. of article/Sample size	Risk of bias tool
Sheraton et al. [17] (2000–2020)	LUCAS and AutoPulse	15 OCHA/18,474	Cochrane Risk of Bias tool Newcastle–Ottawa Scale
Liu et al. [18] (Up to 2019)	LUCAS	6 OCHA/8501	Cochrane Risk of Bias tool
Li et al. 2016[19] (1946–2015)	LDBs (AutoPulse), pistons (LUCAS and Thumper), and Pneumatic vests (vest CPR)	9 OHCA 3 IHCA/11,162	Tool not specified
Khan et al. [20] (Up to 2017)	LUCAS, AutoPulse	6 OHCA 1 Combined/12,908	Cochrane Risk of Bias tool
Couper et al. [21] (1946–2016)	Thumper, Pneumatic Vest, Lifestat (Piston), LUCAS, AutoPulse, Load-distributing band device	9 IHCA-Hospital/689	Cochrane Risk of Bias tool GRADE
Gates et al. [22] (1990–2015)	LUCAS, AutoPulse	5 OHCA/12,206	Cochrane Risk of Bias tool
Bonnes et al. [23] (2000–2014)	LUCAS, AutoPulse	20 OHCA/21,363	Cochrane Risk of Bias tool Newcastle–Ottawa Scale
Zhu et al. [24] (Up to 2019)	LUCAS, AutoPulse, Thumper, Life-Stat	15 OHCA/104,715	Cochrane Risk of Bias tool Newcastle–Ottawa Scale
Tang et al. [25] (Up to 2015)	﻿LUCAS, AutoPulse	5 OHCA/12,510	Cochrane Risk of Bias tool
Wang et al. [26] (1946–2017)	Thumper, Pneumatic Vest, Piston, LUCAS, AutoPulse	7 OHCA 3 IHCA 1 Combined/12,944	Cochrane Risk of Bias tool
Chinag et al. [27] (Up to 2021)	LUCAS and AutoPulse	22 OHCA/85,975	ROBINS-I Cochrane Risk of Bias tool

**Table 2:** Characteristics of the systematic reviews included (continued)

References	Study design	Outcomes and heterogeneity	Conclusion
[17]	6 RCTs 2 Cluster RCTs 5 Retrospective case–control 2 phased prospective cohort studies	ROSC (I^2^ 83%)	Mechanical devices during CPR do not improve ROSC outcomes, even though they improve the quality of CPR
[18]	4 RCT Two non RCT	ROSC (I^2^ 20%) Survival to hospital admission (I^2^ 79%) Survival to hospital discharge (I^2^ 3%) Survival to 30 days (I^2^ 43%)	Mechanical chest compression with a LUCAS device does not improve clinical outcomes in out-of-hospital CA patients compared with manual chest compression
[19]	8 RCT 2 phased prospective cohort trials 1 phased prospective cohort trial 1 descriptive controlled trial	ROSC (I^2^ 83%) Survival to hospital admission (I^2^ 60%) Survival to hospital discharge (I^2^ 71%) Good neurological outcome after hospital discharge (I^2^ 59%)	The ability to achieve ROSC with a mechanical device was inferior to manual chest compression during resuscitation
[20]	7 RCT 3 Cluster RCT 1 Quasi RCT	Survival at 30 days or Hospital discharge(I^2^ NA) Survival to hospital admission ROSC Neurological recovery Visceral damage Sternal or rib fracture Pneumothorax Hematoma formation	CPR with manual compression showed better survival at 30 days or hospital discharge and neurological outcomes than AutoPulse, while manual compression had a similar efficacy profile to LUCAS
[21]	3 RCT 2 Crossover 4 Observational cohort	Survival at 30 days or Hospital discharge (I^2^ 0%) Survival with good neurological outcome (I^2^ NA) ROSC (I^2^ 19%) Physiological outcomes Safety outcomes (I^2^ NA)	Mechanical chest compression devices may improve patient outcomes when used at IHCA
[22]	3 RCT 2 Cluster randomized trials	Survival at 30 days or Hospital discharge (I^2^ 0%) ROSC (I^2^ 49%) Survival with good neurological outcome (I^2^ 68%) Survival to hospital admission (I^2^ 0%)	Mechanical chest compression devices are not superior to manual chest compression
[23]	5 RCT 15 non RCT	Survival to hospital admission (I^2^ 39%) ROSC (I^2^ 78%) Survival to hospital discharge (I^2^ 80%) Good neurologic outcome at discharge. (I^2^ 80%)	Cumulative evidence of RCT data does not support a routine strategy of mechanical CPR to improve clinical outcomes, but Non-RCT studies provide the superiority of mechanical CPR
[24]	9 RCT 6 Cohort Studies	ROSC (I^2^ 87%) Survival to hospital admission (I^2^ 64%) Survival to hospital discharge (I^2^ 70%) Good neurological outcome (I^2^ 82%)	There were no significant differences in resuscitative effects between mechanical and manual chest compression in OHCA patients
[25]	5 RCT	Survival with good neurological outcome to hospital discharge Survival to hospital admission (I^2^ 0%) Survival to hospital discharge (I^2^ 0%) ROSC (I^2^ 0%) Long-term (≥ six months) survival (I^2^ 16%)	Mechanical chest compressions were not associated with better outcomes in OHCA compared with manual chest compressions
[26]	7 RCT 3 Cluster RCT 1 Quasi RCT	Survival to hospital discharge with good neurological function (I^2^ > 50%) ROSC Survival to hospital admission Short-term survival (less than or equal to 30 days) Long-term survival (greater than 30 days) Survival to hospital discharge Sternal or rib fracture hemothorax or pneumothorax Abdominal organ injury	Evidence from RCTs in humans is insufficient to conclude that mechanical CPR is associated with benefit or harm
[27]	7 RCT 15 non RCT	ROSC (I^2^ 89%) Survival to hospital admission (I^2^ 85%) Survival to Discharge (I^2^ 86%) Survival to Discharge with good neurological outcome (I^2^ 78%)	Prehospital use of mechanical CPR devices may benefit adult OHCA patients to achieve ROSC and survival to hospital admission

The sample size for each SR ranged between 689 and 104,715 patients. The SR conducted by Couper et al. [21] was the smallest (n = 689), and Zhu et al. SR [24] was the largest (n = 104,715). There were 46 unique original studies included in the UR, of which 14 were RCTs (Fig. 1). Three studies were included in most SRs [6–8], Hallstrom et al. [12], Wik et al. [32], and Axelsson et al. [33] in nine, eight, and seven SRs, respectively. Only three studies [34–36] were published before 2003, when neither LUCAS nor AutoPulse were available. Axelsson et al. [33] was the only study used in more than 6 SRs and was not an RCT. Only four SRs explicitly excluded traumatic cardiac arrest. Studies used in the individual SRs can be found in Appendix file. A complete list of randomized and non-randomized studies can be found in Appendix file.

### Outcomes

All the included SRs reported ROSC as a primary (six SRs) or secondary (five SRs) outcome. Four SRs reported survival to hospital discharge or survival at 30 days as their primary outcome, and six reported survival to hospital admission as the primary outcome. Three SRs reported survival to hospital admission as primary, and six reported survival with good neurological outcomes. Survival with good neurological outcomes was reported in three SRs as primary and six of them as secondary outcomes. A summary of the results can be found in Table 3.

**Table 3:** Summary of systematic reviews in the UR

	ROSC	Survival to hospital admission	Survival to hospital discharge OR 30 days	Neurological recovery
Sheraton et al. [17]	No^a^	NA	NA	NA
Liu et al. [18]	No	No	No	NA
Li et al. [19]	No	No	No	No
Khan et al. [20]	No	No	No	No
Couper et al. [21]	Improved^b^	NA	Improved	NA
Gates et al. [22]	No	No	No	No
Bonnes et al. [23]	Partially^c^	Partially	No	No
Zhu et al. [24]	No	No	No	No
Tang et al. [25]	No	Negative effect^e^	No	No
Wang et al. [26]	NA^d^	NA	Negative effect	Negative effect
Chiang et al. [27]	Partially	Partially	No	No

^a^No improved outcome in the mechanical CPR group

^b^Mechanical CPR group showed improvement

^c^Mechanical CPR group showed partial improvement

^d^Result not available

^e^Mechanical CPR group showed adverse effects

#### ROSC

**Fig. 2:**
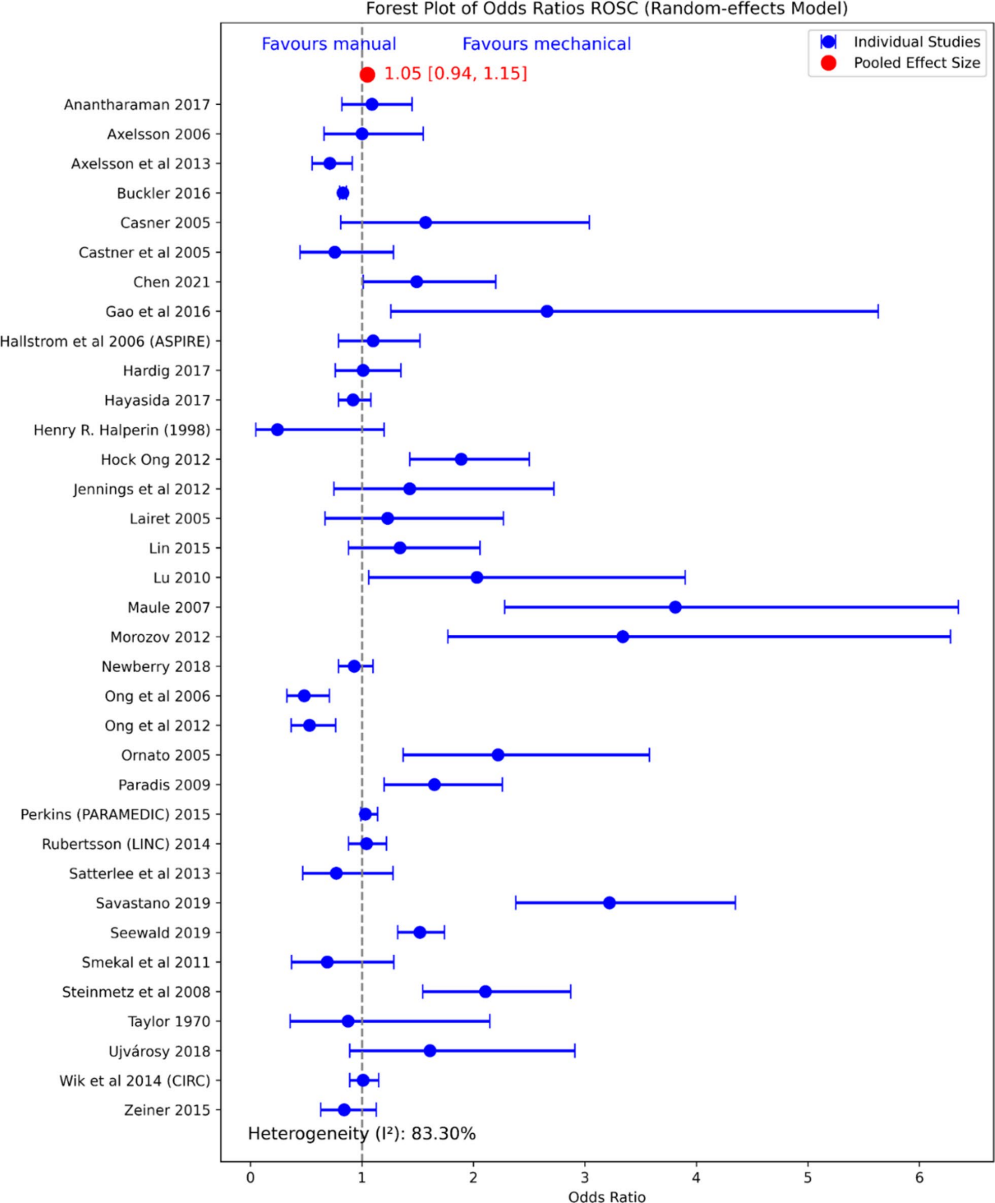
Umbrella review Forest plot for ROSC

Ten out of the 11 SRs pooled the results for ROSC. Wang et al. [26] did not pool the results, accounting for the heterogeneity. Seven out of the 10 SRs showed no improvement in ROSC by using mechanical CPR. In contrast, Chiang et al. [27], Bonnes et al. [23], and Couper et al. [21] showed improved ROSC by mechanical CPR [22, 23, 27]. In Chiang et al. and Bonnes et al. SRs, the subgroup analysis including solely RCT studies, showed no improvement over manual CPR. Bonnes et al. [23] included seven abstracts in their SR, further reducing the quality of the evidence. Couper et al., with the smallest sample size (n = 689), reported low quality of the evidence analyzed for all outcomes. The pooled effect of ROSC [Odds ratio 1.05 (0.94–1.15)] from all the individual original studies with available data included in the UR after removing duplicates and outliers can be found in Fig. 2. High heterogeneity (I^2^ 83%) was observed between the studies.

#### Survival to hospital admission

Eight SRs on OHCA reported a pooled effect of mechanical CPR on survival to hospital admission. Wang et al. [26] reported this outcome from individual papers but did not pool the results because of the heterogeneity effect. Five reported no superiority to mechanical CPR, while Tang et al. [25] reported worse outcomes. Chiang and Bonnes et al. reported a significant improvement but failed to show this effect in a subgroup analysis including only RCT [23, 27].

#### Survival to hospital discharge or 30 days

**Fig. 3:**
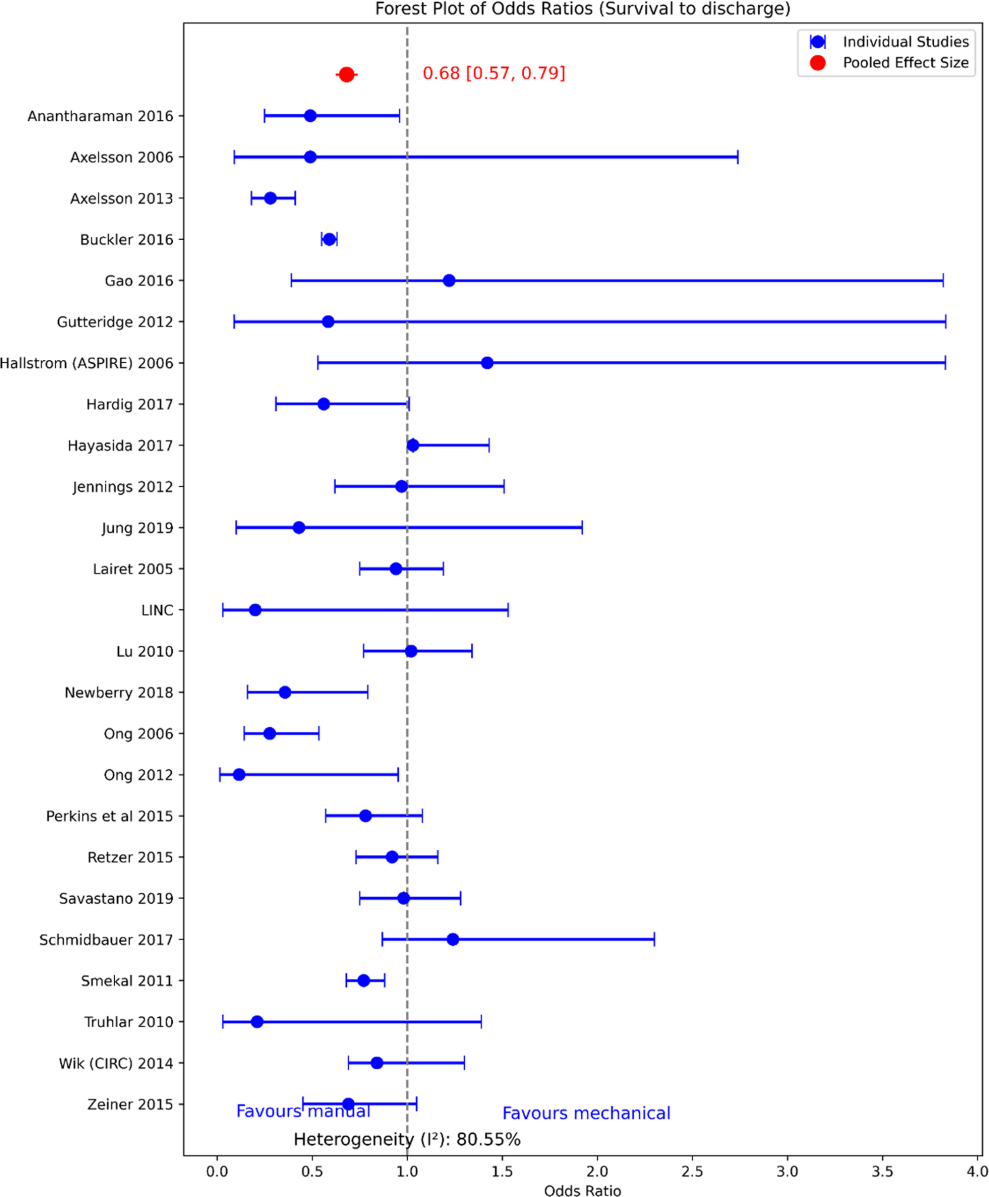
Umbrella review for survival to hospital discharge

Of the eleven SRs analyzing survival to hospital discharge or 30 days, nine reported no superiority for mechanical CPR. Wang et al. reported a worse outcome to mechanical CPR, while Bonnes et al. reported improvement [23, 26], annulled when conducting the subgroup analysis, including only RCTs. The pooled effect of survival to hospital discharge or 30 days [odds ratio 0.68 (0.57–0.79)] from the individual studies with available data that is included in the SRs after removing duplication and outliers is available in Fig. 3. High heterogeneity (I^2^ 81%) between study was observed between the studies.

#### Neurological recovery

Only eight SRs reported neurological outcomes of any sort. None of them reported a better effect after using mechanical CPR. Wang et al. [26] reported a worse outcome, which was based on a single study [15].

## Methodological quality

Table 4 summarizes the methodological quality of the individual SRs included. Unfortunately, every SR, except one [26], included at least two critical flaws, which makes the confidence in the results “critically low.” Only three SRs registered the protocols before performing the analysis [21, 26, 27], and only two SRs provided a list of the excluded studies [17, 26]. Most SRs had at least three critical flaws [17–25], while others had five crucial flaws. Thus, it had the lowest methodological quality [23]. Ten SRs performed metanalyses; however, only two justified combining the data in the meta-analysis based on the appropriate methods for statistical combination of results as per AMSTAR-2 Tool [25, 27]. Only one SR did not perform a meta-analysis [26], for which the AMSTAR-2 questions 11, 12, and 15, as shown in Table 4, were not applicable. Li et al. [19] did not use a standard assessment tool to evaluate the methodological quality. Instead, they used four measures (description of randomization, allocation concealment, description of withdrawals, and binding outcome assessment). All other SRs used the Cochrane risk of bias tool for RCTs, while Sheraton et al. [17], Bonnes et al. [23], and Zhu et al. [24] used the Newcastle–Ottawa Scale for non-RCTs. Couper et al. [21] used the Cochrane risk of bias assessment for RCTs and the Grading of Recommendations Assessment, Development, and Evaluation (GRADE) tool to evaluate the risk of biases in observational studies. Tang et al. [25], Wang et al. [26], and Chiang et al. [27] used the GRADE system to measure the quality of outcomes along with the Cochrane risk of bias tool.

**Table 4:** Quality assessment of included SRs based on AMSTAR-2 Tool. ROB is a risk of bias

Q. no.	Questions	Sheraton et al. 2021	Liu et al. 2019	Li et al. 2016	Khan et al. 2018	Couper et al. 2016	Gates et al. 2015	Bonnes et al. 2016	Zhu et al. 2019	Tang et al. 2015	Wang et al. 2018	Chiang et al. 2022
1	Research questions and inclusion criteria included PICO	Yes	Yes	Yes	Yes	Yes	Yes	Yes	Yes	Yes	Yes	Yes
2	Protocol registered before the commencement of the review	No	No	No	No	Yes	No	No	No	No	yes	Yes
3	Explanation of the selection of the studies for inclusion	Yes	No	No	No	No	No	No	No	Yes	No	Yes
4	Adequacy of literature search	Yes	Yes	Yes	Yes	Yes	Yes	Yes	Yes	Yes	Yes	Yes
5	Study selection performed in duplicate	Yes	Yes	Yes	Yes	Yes	Yes	Yes	Yes	Yes	Yes	Yes
6	Data extraction performed in duplicate	Yes	Yes	Yes	Yes	Yes	Yes	Yes	Yes	Yes	Yes	Yes
7	Justification for excluding studies	Yes	No	No	No	No	No	No	No	No	No	No
8	Studies described in adequate detail	Yes	Yes	Yes	Yes	Yes	Yes	Yes	Yes	Yes	Yes	Yes
9	ROB from individual studies being included in the review	Yes	Yes	Yes	Yes	Yes	Yes	Yes	Yes	Yes	Yes	Yes
10	Reporting sources of funding for studies included in the review	No	No	No	No	Yes	No	No	No	Yes	No	No
11	Appropriateness of meta-analytical methods	No	No	No	No	No	No	No	No	Yes	NA	Yes
12	Assessment of the potential impact of ROB on results	No	Yes	No	Yes	No	Yes	No	Yes	Yes	NA	No
13	Consideration of ROB when interpreting the results of the review	No	Yes	Yes	Yes	Yes	Yes	No	Yes	Yes	Yes	No
14	Heterogeneity satisfactorily explained and discussed	Yes	No	Yes	Yes	Yes	Yes	No	No	Yes	Yes	Yes
15	Assessment of presence and likely impact of publication bias	Yes	Yes	Yes	No	No	No	No	Yes	No	NA	Yes
16	Reporting of potential conflicts of interest and review funding	Yes	Yes	Yes	Yes	Yes	Yes	No	Yes	Yes	Yes	Yes

## The new additional SR

**Fig. 4:**
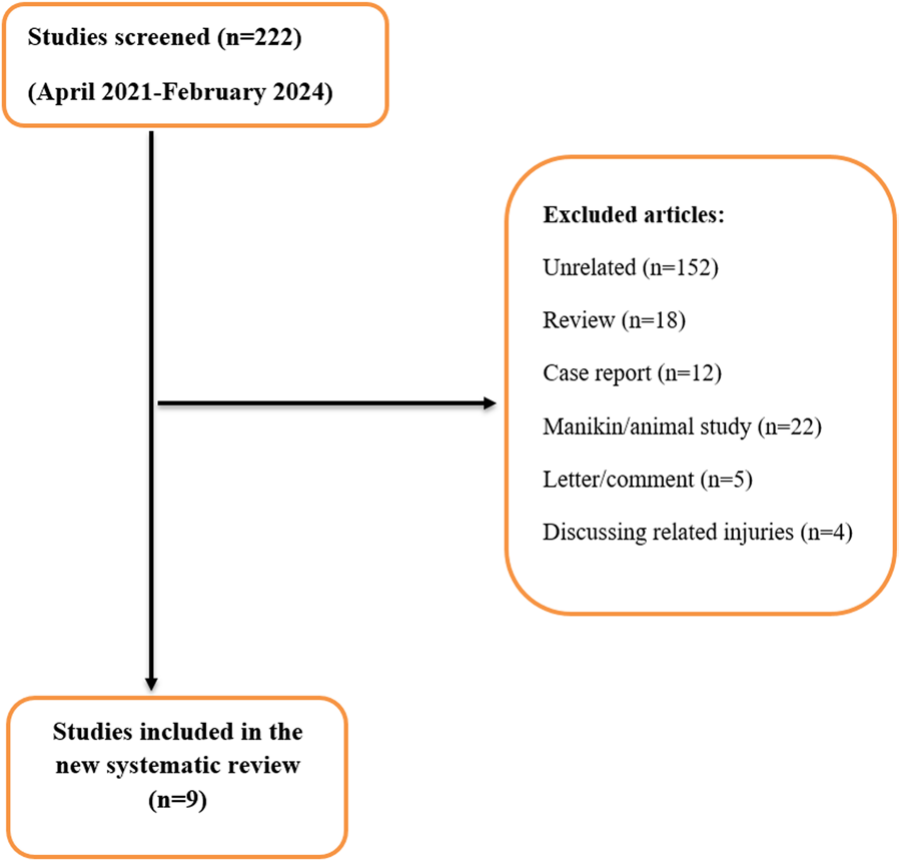
PRISMA flow chart for the new systematic review (April 2021–February 2024)

Following similar conditions used in the UR, we identified 182 individual studies between April 2021 and February 15, 2024, to be included in the new SR (Fig. 4). Two studies were not included in any of the SRs of the Umbrella review, so we involved them in the new SR [37, 38]. Nine papers were eligible and selected for the new SR analysis. All the excluded references are available in Appendix file.

### Characteristics of original studies of the new SR

Nine studies were included in the analysis [37–45], of which none were RCTs but retrospective observational studies. Seven of them were OHCA studies, and Two were IHCA. Of the 134,624 patients analyzed, 111,446 were IHCA and 42,503 OHCA. Kim et al. study [42] was the largest (n = 20,170) OHCA study, whereas Crowley et al. studied 111,143 IHCA cases. Three studies were conducted in South Korea, two in the USA, and one in Germany, Thailand, Italy, and Turkey. Kim et al. [40] and Crowley et al. [45] did not mention the specific CPR device, but in all the other studies, LUCAS was at least one of the devices analyzed. Three studies compared LUCAS to manual CPR and two compared different mechanical devices. Kim et al. [38] compared AutoPulse vs. LUCAS vs. Thumpar, while Primi et al. [43] compared LUCAS, AutoPulse, and Easypulse. The IHCA study conducted by Sener et al. [41] was the only study including trauma patients (n = 18). Two studies did not comment on whether traumatic cardiac arrest was included, while the other studies explicitly mentioned trauma as an exclusion criterion. A summary of the characteristics of the studies can be found in Tables 5,6.

**Table 5:** Characteristics of the original studies included in the systematic review

References and duration	Study setting/sample size	Country	Study design	Intervention/device type
Gässler et al. [39] (2007–2020)	OHCA/4851	Germany	Retrospective observational	LUCAS, AutoPulse, Corplus
Kim et al. [40] (2020–2021)	OHCA/842	South Korea	Retrospective study	The device type was not mentioned
Şener et al. [41] (2016–2018)	IHCA/303	Turkey	Retrospective Cohort	LUCAS
Min et al. [37] (2017–2020)	OHCA/3230	South Korea	Retrospective observational	Easypulse, LUCAS
Tantarattanapong et al. [42] (2017–2019)	OHCA/227	Thailand	Retrospective chart review	LUCAS
Kim et al. [38] (2012–2016)	OHCA/20170	South Korea	Retrospective observational	AutoPulse, Thumpar, Lucas
Primi et al. [43] (2015–2022)	OHCA/12901	Italy	Retrospective cohort	Easypulse, LUCAS, AutoPulse
Mastenbrook et al. [44] (2011–2017)	OHCA/282	USA	Retrospective observational	LUCAS
Crowley et al. [45] 2011–2019	IHCA/111143	USA	Retrospective study	Mechanical 2% vs 98% manual CPR. The device type was not mentioned

**Table 6:** Characteristics of the original studies included in the systematic review

References	Outcomes	Conclusion
[39]	30 Day survival ROSC Good neurological outcome	Mechanical devices are not associated with better survival when used during transport. Devices are associated with better survival in prolonged resuscitation but worse survival when a fibrinolytic is used
[40]	Survival at discharge Good neurological outcome at discharge ROSC	This study found no significant differences in survival rates and neurological outcomes between mechanical CPR and PPE-equipped manual CPR in the ED setting
[41]	ROSC 7/30-day survival	The use of piston-based mechanical CC devices in ED may be beneficial
[37]	Neurological outcome Survival to discharge	Prehospital mechanical chest compression device use in OHCA was associated with poorer neurologic outcomes and survival to discharge
[42]	ROSC Survival to hospital admission Good neurological outcome	Mechanical chest compression was not associated with sustained ROSC
[38]	Survival at hospital discharge ROSC	The mechanical CPR devices largely led to similar survival to discharge as that of manual CPR in OHCA patients
[43]	ROSC 30-day mortality	Mechanical chest compressors could increase the ROSC rate, especially in prolonged resuscitation. The load-distributing-band device was the only mechanical chest able to affect 30-day survival favorably
[44]	ROSC	There is no difference in prehospital ROSC rates among adult non-traumatic cardiac arrest patients when comparing mechanical-assisted and manual-only CPR
[45]	Survival to discharge ROSC	Mechanical CPR and survival to discharge (OR 0.49 in prolonged IHCA, 0.74 in 5–20 min IHCA, 0.51 in non-shockable rhythms, and 0.53 in shockable rhythms). Mechanical CPR and ROSC (adjusted OR 0.68), *p* < 0.01 for all

### Results of the original studies used in the new SR

Eight of the nine included studies discussed ROSC as an outcome. Six concluded that mechanical CPR does not improve ROSC. Gässler et al. [39] studied OHCA and showed a better ROSC and 30-day survival for prolonged (> 45 min) CPR utilizing mechanical rather than manual CPR. However, the authors reported that ROSC and 30-day survival were significantly worse when a device was used in addition to fibrinolytic agents. For those who were transported with ongoing resuscitation, the authors found similar 30-day survival but better ROSC with manual CPR. Primi et al. [43] showed better results for ROSC with a propensity-score-based analysis, but the raw logistic regression model showed no significant differences. Only one study on OHCA reported survival to hospital admission, which showed no superiority of mechanical CPR. Six studies reported survival to hospital discharge or 30 days, of which four reported no improvement with mechanical CPR. Gässler et al. reported “an improvement” for the prolonged CPR subgroup. Primi et al. compared propensity score matched groups, which showed a significantly better performance for mechanical CPR, specifically AutoPulse. Crowley et al. [45] conducted the largest IHCA study; however, mechanical CPR was used in only 2% of the patients. A summary of the results of the original studies can be found in Table 7.

**Table 7:** Result summary of original studies included in the new systematic review

	ROSC	Survival to hospital admission	Survival to hospital discharge OR 30 days	Neurological recovery
Gässler et al. [39]	Partially^a^	NA	Partially	Partially
Kim et al. [40]	No	NA	No	No
Sener et al. [41]	No	NA	No	NA
Min et al. [37]	NA	NA	No	No
Tantarattanapong et al. [42]	No	No	No	No
Kim et al. [38]	No	NA	No	NA
Primi et al. [43]	Partially	NA	Partially	NA
Mastenbrook et al. [44]	No	NA	NA	NA
Crowley et al. [45]	No	NA	No	NA

Partially = Result is partially supporting mechanical CPR. No = Result not supporting mechanical CPR. NA = Result not available

### Use of mechanical CPR according to gender and age

The use of mechanical CPR based on the patient's gender and age is presented in Table 8. Only four studies showed a significantly higher utility of mechanical CPR in male patients. Notably, all the studies that showed a significant gender difference had a higher patient population. Two studies showed that younger patients significantly received more mechanical CPR compared to older patients. Otherwise, there were no significant differences based on gender and age regarding the type of CPR. Moreover, none of the studies explicitly comment on the outcomes based on the patient's gender or age.

**Table 8:** Gender and age distribution of mechanical and manual CPR groups

Study	Mechanical CPR		Manual CPR		*P* value*	Mechanical CPR	Manual CPR	*P* value*
	Total	M/F (%)	Total	M/F (%)		Age distribution		
Gässler et al. [39] (a)	2143	76.8/23.2	2708	67.6/32.4	0.01	62.7 ± 15.3	63.3 ± 20.4	0.18
Gässler et al. [39] (b)	374	70.6/29.4	3546	65.7/34.3	0.06	64.9 ± 14.6	67.1 ± 17.2	0.30
Gässler et al. [39] (c)	468	76.1/23.9	1638	75.2/24.8	0.69	59.7 ± 13.5	61.0 ± 13.9	0.08
Kim et al. [40]	1331	66/34	421	67/33	0.70	74.0 (59.0, 82.0)	72.0 (62.0, 80.0)	0.30
Şener et al. [41]	180	56.1/43.9	123	47.2/52.8	0.13	75.0 (62.3, 84.0)	77.0 (65.0, 86.0)	0.24
Min et al. [37]	2119	64.7/35.3	1111	61/39	0.04	75.0 (63.0, 81.0)	75.0 (62.0, 83.0)	0.43
Tantarattanapong et al. [42]	34	73.5/26.5	193	61.7/38.3	0.26	59.0 (50.8, 66.8)	68.0 (54.0, 79.0)	0.01
Kim et al. [38] (d)	671	66.3/33.7	671	65/35	0.65	69.0 (57.0, 78.0)	69.0 (57.0, 78.0)	0.95
Kim et al. [38] (e)	305	67.2/32.8	305	60.7/39.3	0.11	71.0 (56.0, 78.0)	69.0 (56.0, 78.0)	0.79
Kim et al. [38] (f)	149	67.8/32.2	149	64.4/35.6	0.62	70.0 (56.0, 78.0)	68.0 (56.0, 79.0)	0.71
Primi et al. [43]	2405	72.6/27.4	10,496	56.8/43.2	0.01	66.0 (55.0, 76.0)	80.0 (69.0, 87.0)	0.01
Mastenbrook et al. [44]	79	58.2/41.8	110	62.7/37.3	0.37	66.24 ± 16.62	65.81 ± 15.10	0.44
Crowley et al. [45]	2232	62.7/37.3	108,911	58.9/41.1	0.01	70 (59, 78)	68 (57, 77)	0.06

*Between-group differences concerning gender were assessed using the chi-square test or Fisher's exact test, and the two-sample t-test or Mann–Whitney U test was used to analyze age

(a) On-going CPR, (b) prolonged CPR, (c) fibrinolytic therapy used, (d) Autopulse, (e) LUCAS and (f) Easypulse device

### Initial cardiac arrest rhythms (shockable vs. non-shockable)

All included studies reported incidents of shockable rhythm based on the type of CPR, but few reported their outcome based on the type of CPR. Out of 12,232 patients receiving mechanical CPR, 2723 had shockable rhythm, and out of 122,032 patients receiving manual CPR, 19,179 had shockable rhythm. The detailed incidence data is available in Appendix file. Only Min et al. [37] reported neurological recovery based on the initial rhythm and type of CPR, which shows a negative effect with mechanical CPR. The result needs to be considered cautiously as the patient population is very small (only eight patients had good neurological outcomes). Gässler et al.'s study was the only one that reported on prolonged CPR [39].

### IHCA versus OHCA

Only two studies [41, 45] were conducted in the IHCA setting. Even though Crowley et al. [45] was the largest study, it only had 2% (n = 2232) of patients in the mechanical CPR group. Seven studies reported OHCA cases, of which only one reported survival at hospital admission, and four reported neurological outcomes. A pooled result of odds ratio from the reported studies is available in Table 9.

**Table 9:** Sensitivity analysis based on the initial rhythm, location of cardiac arrest, and duration of CPR

Variable	Mechanical CPR	Manual CPR	Outcome (odds ratio)
	Incidents (n/%)	Incidents (n/%)	
Shockable rhythm	2723/22.3% [37–45]	19,179/15.7% [37–45]	a. 0.84 (0.70–1.03) [37, 38, 45] b. 1.48 (0.91–2.41) [38] c. 0.15 (0.06–0.33) [37]
Non Shockable	6040/85.1% [37, 38, 40–42, 44, 45]	16,936/15.1% [37, 38, 40–42, 44, 45]	a. 0.50 (0.41–0.60) [37, 45] c. 0.17 (0.05–0.63) [37]
IHCA	2412/100% [41, 45]	109,034/100% [41, 45]	a. 1.49 (0.59–3.78) [41, 45] b. 0.70 (0.47–1.06) [41, 45]
OHCA	9820/100% [37–40, 42–44]	12,998/100% [37–40, 42–44]	a. 0.87 (0.41–1.84) [37–40, 42, 43] b. 0.68 (0.63–0.75) [38–40, 42–44] c. 0.66 (0.41–1.06) [37, 39, 40, 42] d. 0.11 (0.02–0.45) [42]
Prolonged CPR	374/12.5% [39]	3546/44.9% [39]	a. 2.72 (1.48–3.48) [39] b. 1.77 (1.43–2.19) [39] c. 2.29 (1.37–3.81) [39]

a = Survived until discharge, b = ROSC, c = Neurological recovery, d = Survival at hospital admission

### Type of device in the mechanical CPR (PD vs. LDB)

Out of the nine studies, only six explicitly had data on the type of CPR device (Table 10). Of these, 4313 patients received mechanical CPR from piston-based devices (LUCAS, Thumpar, Easypulse) and 1387 received CPR from LDB based devices (Autopulse). Even though the average outcome percentage shows slight superiority for LDB, the amount of data is limited.

**Table 10:** Outcomes based on the type of mechanical CPR device

Study	Piston based (PD)		LDB	
	Total	Outcome	Total	Outcome
Sener et al. [41]	180	a. 19 (10.6%) b. 75 (41.7%)	NA	NA
Min et al. [37]	2119	a.31 (1.5%) c. 11 (0.5%)	NA	NA
Tantarattanapong et al. [42]	34	a. 0 (0%) b.3 (8.8%) c. 0 (0%) d. 2 (5.9%)	NA	NA
Kim et al. [38]	474	a. 14 (3.1%) b. 103 (22.7%)	671	a. 33 (4.9%) b. 203 (30.3%)
Primi et al. [43]	1426	a. 55 (3.9%) b. 157 (11%)	716	a. 67 (9.4%) b. 156 (21.8%)
Mastenbrook et al. [44]	80	b. 25 (31.25%)		
Total	4313	a. 119 (5.3%) b. 363 (16.5%) c. 11 (0.5%)	1387	a. 100 (7.2%) b. 359 (25.9%)

*LDB* load distributing band, *NA* not applicable, a = Survived until discharge, b = ROSC, c = Neurological recovery, d = Survival at hospital admission

### Methodological quality of studies included in the new SR

None of the included studies stated how the choice of using mechanical or manual CPR was made (Table 11). Even though most of the studies matched the exposed (Mechanical CPR) and non-exposed (Manual CPR) cohort or adjusted for the confounders in the analysis, Sener et al. [41] and Mastenbrook et al. [44] failed to do so. Tantarattanapong et al. [42] compared the outcomes without adjusting for the confounding factors, but a multivariate logistic regression model was used to find the associated factors with the ROSC. Crowley et al. [45] generated a propensity score with the mode of CPR as the dependent variable and patients, hospital level, and arrest characteristics as independent variables. However, the authors performed sensitivity analysis and multiple imputations for missing data in subanalyses.

**Table 11:** Newcastle–Ottawa Scale scoring for the observational studies

Study	Selection	Comparability	Outcome	Score
Gässler et al. [39]	2	2	3	7/9
Kim et al. [40]	3	2	3	8/9
Sener et al. [41]	3		3	6/9
Min et al. [37]	3	2	1	6/9
Tantarattanapong et al. [42]	3	1	3	7/9
Kim et al. [38]	3	2	3	8/9
Primi et al. [43]	2	2	3	7/9
Mastenbrook et al. [44]	2		3	5/9
Crowley[45]	2	2	3	7/9

### GRADE assessment and outcomes of the new SR

**Fig. 5:**
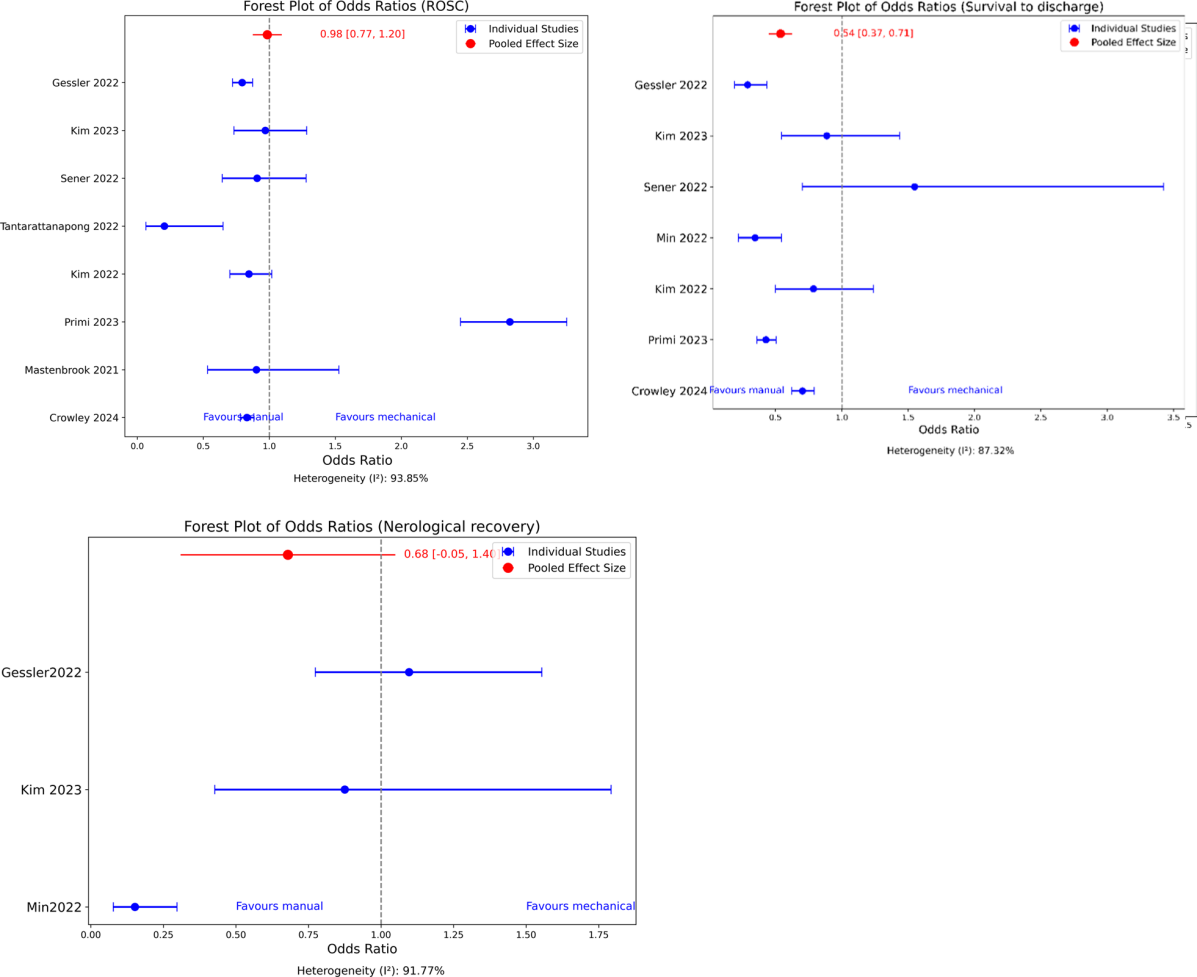
Odds ratio and 95% confidence intervals for the Outcomes of the studies included in the new systematic review: Restore spontaneous circulation (ROSC) (upper left panel) Survival to hospital discharge or 30 Days (upper right panel), Neurological recovery (lower panel)

The GRADE assessment revealed that overall confidence in the entire outcomes were very low (Table 12). We downgraded for risk of bias as most of the individual studies had low or moderate methodological quality. Inconsistency was downgraded due to the high heterogeneity and the non-consistent direction of effect. The indirectness was absent as all the studies directly compared manual and mechanical CPR. The wide confidence intervals of the odds ratio contributed to the imprecision, and publication bias was not observed (Fig. 5). None of the three rating up criteria of the GRADE tool were applicable to the studies included. The assessment of survival to hospital admission was reported only in one study; hence, it was excluded from calculating the certainty of evidence.

**Table 12:** Certainty assessment GRADE too

Outcome	Risk of bias	Inconsistency	Indirectness	Imprecision	Publication bias	Overall certainty of evidence
ROSC	Very serious^a^	Very serious^b^	Not serious	Serious^c^	None	⊕ OOO Very low
Survival to hospital discharge OR 30 days	Very serious	Very serious	Not serious	Very serious	None	⊕ OOO Very low
Neurological recovery	Very serious	Very serious	Not serious	Very serious	None	⊕ OOO Very low

^a^Most of the included observational studies were low or moderate quality

^b^Very high heterogeneity between studies

^c^Wide 95% confidence intervals of individual studies

## Discussion

Many publications have studied 'mechanical versus manual CPR' in the last two decades, including 11 systematic reviews. This is the first umbrella review on this topic. In this comprehensive analysis, we aimed to conduct a dual-faceted SR, which analyzed the SRs published to date and added an original SR on the evidence published after the last SR, thus all publications to date. This unique approach aimed to provide a panoramic view of all existing evidence on the effectiveness of mechanical devices in CPR. The combination of all evidence failed to demonstrate that mechanical CPR improves ROSC, survival to hospital admission, survival to hospital discharge or 30 days, or survival to hospital discharge with good neurological function compared to manual CPR. However, the heterogeneity, quality, and biases of the SR and studies should be considered. Furthermore, concerning mechanical CPR, there are many factors not clearly explained in the studies [45], such as at what time during CPR the device was applied, the time needed to attach the device, the type of device, the patient and hospital-level variables and the bystander team, protocol, and training levels.

One SR [46] strongly contradicts the findings of the present global analysis. In Westfall et al. SR, most individual studies were non-randomized, and Wang et al. later noted that all authors had a financial relationship with manufacturers of mechanical CPR devices. Wang et al. [26] was the only updated study which did not perform a meta-analysis due to high clinical heterogeneity.

Gates et al. [22] and Tang et al. [25] published their SRs in 2015, including the same original papers, and reached the same conclusion that mechanical CPR does not improve outcomes. Tang et al. reported worse outcomes on survival to hospital admission for mechanical devices.

The only IHCA SR included in the UR was conducted by Couper et al. [21]. This study showed an improved outcome after mechanical CPR. However, considering its sample size (n = 689), it should be interpreted cautiously. The median sample size for the UR was 12,908 patients. Furthermore, Couper’s SR included studies from 1978 to 2010, spanning over three decades. Moreover, all outcomes measured in this SR were of very low quality.

None of the OHCA SRs showed better outcomes to mechanical CPR when only RCTs were analyzed. Chiang et al. [27] and Bonnes et al. [23] showed improvement in short-term survival (ROSC and survival to hospital admission) when pooling data from non-RCTs. Both studies used 15 non-RCTs in their analysis, which is the highest number of publications among all the SRs considered. Of note, 7 of the non-RCTs were abstracts in the Bonnes et al. [23] study.

Lameiger et al. [47] published an SR in 2015 about mechanical CPR in IHCA patients. Nine out of 14 studies included were either case studies or case reports. None of the individual studies appropriately compared mechanical CPR. The study had 89 patients who underwent mechanical CPR using the LUCAS device, of which 17 were case reports or series. The study concluded that early use of mechanical CPR could improve patient outcomes, but the evidence supporting the conclusion was low.

We analyzed the methodological quality of the SRs using the AMSTAR-2 tool. All the included SRs were rated as “critically low quality” except for the Cochrane review [26], which was of “moderate quality.” Despite the authors of the Cochrane review mentioning that the protocol was published in the third issue of the 2008 version, it was not accessible online. Most of the SRs failed to publish a protocol, provide a list of excluded studies, and state the use of appropriate methods for the statistical combination of results.

We have also systematically analyzed all the original papers published after March 2021 to understand the direction of the most recent studies that were not included in any SRs period. Out of 9 studies included, and four different outcomes, 7 showed no superiority for mechanical CPR. Gässler et al. [39] showed more ROSC, survival to hospital discharge or 30 days, and survival to hospital discharge with good neurological function only for the “prolonged” CPR subgroup. The study, however, did not show any significant benefit after mechanical CPR in any other subset. Primi et al. [43] was the second study showing any significantly better outcome for mechanical CPR. When analyzed without the propensity score matching, the study reported a negative effect for both LUCAS and EasyPulse in the raw logistic regression model. Even though few studies showed a significant difference in the use of mechanical and manual CPR groups according to gender or age, there was no information on the outcomes based on these two variables. All studies reported the type of initial rhythm of patients, but only a few reported the outcome based on the type of rhythm. Only one study [39] reported data on prolonged CPR. When comparing the types of devices used for mechanical CPR, LDB-based devices showed a higher percentage of ROSC and survival until discharge. The NOS-based quality assessment for the included studies showed that none of the observational studies explicitly explained the choice between mechanical and manual CPR. The GRADE assessment showed that the overall quality of evidence for all outcomes was very low.

## Limitations

We pooled the data irrespective of the type of devices (e.g., LUCAS or AutoPulse) or their updated versions, the design of the studies included (RCTs or non-RCTs), and whether they were used for in-hospital or OHCA cases, which can lead to clinical heterogeneity. Due to the nature of the intervention, no care providers were blinded by the individual RCTs. There was an overlapping of studies included in the umbrella review, which might influence the overall conclusion driven by these SRs and limited us to attempting an overall meta-analysis for the UR. However, after removing the duplicated and outliers’ studies used in the UR, we showed the pooled effect for some of the outcomes of interest. We did not consider the use of mechanical CPR in exceptional circumstances as an objective of our study. We also did not conduct an analysis based on the etiology of cardiac arrest due to the unavailability of data from the SRs that focused on outcomes. Future research should prioritize investigating the impact of mechanical versus manual CPR across different etiologies of cardiac arrest to provide more nuanced insights into optimal resuscitation strategies for specific patient populations. Even though we analyzed how mechanical CPR devices were used differently according to age and gender, we could not conduct the same for the UR due to the unavailability of the data and the fact that CPR outcomes were not based on age or gender. The latter observation needs more investigation to address the efficacy of either model of CPR on the outcomes based on gender and age. In umbrella reviews, the quality of each SR remains a challenge [48]. However, the current analysis meticulously addresses this issue and adds a new, up-to-date SR. Lastly, the GRADE assessment was not applied to the survival to hospital admission outcome as it was only reported in one study in the new systematic review. In addition, we don’t address whether these resuscitation outcomes were applied to shockable or non-shockable scenarios in some studies/SR and whether they were randomized/distributed equally between the groups. These studies did not have a well-described indication or protocol for using mechanical over manual CPR.

However, one study described the potential benefit of using mechanical CPR during the COVID-19 pandemic compared to manual CPR while wearing personal protective equipment [40]. The study did not find significant differences between the two methods concerning survival and neurological outcomes; however, the authors did not comment on the team and patients’ safety among the two methods of CPR. Mechanical CPR can play a role in countries where an ambulance has to ride long distances before it reaches a hospital. Although an umbrella review acts as a bird’s-eye view, saves research resources, identifies SR gaps, informs future research recommendations, and compares evidence between interventions and associations, it still suffers from the primary studies' quality problems and unsolved biases [49]. After writing and submitting this UR, one SR on the manual vs mechanical CPR during OHCA was published after February 15, 2024 [50]. This SR showed no significant differences between the two types of CPR concerning the ROSC, survival to hospital discharge, and 24-h, and 30-day survival. However, the poorer neurological outcome was more evident after the mechanical CPR.

## Conclusion

Given the significant heterogeneity and methodological limitations, the pooled analysis of the UR and the new SR of the studies published after the last SR  did not provide enough evidence to support a superiority of the mechanical CPR over manual CPR. Only a few low-quality SRs indicated a superiority over manual CPR, and none of the RCT subgroup analyses showed an improvement of outcomes. However, mechanical CPR could be an alternative in selected situations where manual CPR cannot be performed appropriately. High-quality, large-scale RCTs are required to support the UR findings and the SR sub-analyses.

## Supplementary Information

Supplementary Material 1.

Supplementary Material 2.

## Data Availability

All relevant data is given in this manuscript, as well as in tables and appendixes. The protocol is registered and available on PROSPERO ID: CRD42024537182.
