# Supplementary figures and images for: Mechanical versus manual cardiopulmonary resuscitation (CPR): an umbrella review of contemporary systematic reviews and more
Source: Crit Care. 2024 Jul 30;28:259. doi: 10.1186/s13054-024-05037-4 (PMC11290300; doi:10.1186/s13054-024-05037-4)

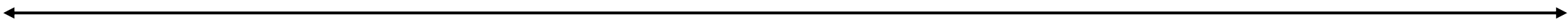

Umbrella review

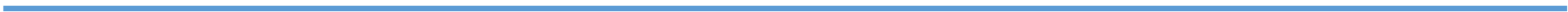

2014 Jan 15

2021 March

2024 Feb 15

New Systematic Review

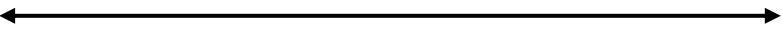

Supplement: Supplementary file 2 — Supplementary Material 2. [file 13054_2024_5037_MOESM2_ESM.pdf]
